# An Immune-Related Long Noncoding RNA Signature as a Prognostic Biomarker for Human Endometrial Cancer

**DOI:** 10.1155/2021/9972454

**Published:** 2021-12-10

**Authors:** Ziwei Wang, Yan Liu, Jun Zhang, Rong Zhao, Xing Zhou, Hongbo Wang

**Affiliations:** Department of Obstetrics and Gynecology, Union Hospital, Tongji Medical College, Huazhong University of Science and Technology, Wuhan 430022, Hubei, China

## Abstract

**Background:**

Endometrial cancer is among the most common malignant tumors threatening the health of women. Recently, immunity and long noncoding RNA (lncRNA) have been widely examined in oncology and shown to play important roles in oncology. Here, we searched for immune-related lncRNAs as prognostic biomarkers to predict the outcome of patients with endometrial cancer.

**Methods:**

RNA sequencing data for 575 endometrial cancer samples and immune-related genes were downloaded from The Cancer Genome Atlas (TCGA) database and gene set enrichment analysis (GSEA) gene sets, respectively. Immune-related lncRNAs showing a coexpression relationship with immune-related genes were obtained, and Cox regression analysis was performed to construct the prognostic model. Survival, independent prognostic, and clinical correlation analyses were performed to evaluate the prognostic model. Immune infiltration of endometrial cancer samples was also evaluated. Functional annotation of 12 immune-related lncRNAs was performed using GSEA software. Prognostic nomogram and survival analysis for independent prognostic risk factors were performed to evaluate the prognostic model and calculate the survival time based on the prognostic model.

**Results:**

Twelve immune-related lncRNAs (ELN-AS1, AC103563.7, PCAT19, AF131215.5, LINC01871, AC084117.1, NRAV, SCARNA9, AL049539.1, POC1B-AS1, AC108134.4, and AC019080.5) were obtained, and a prognostic model was constructed. The survival rate in the high-risk group was significantly lower than that in the low-risk group. Patient age, pathological grade, the International Federation of Gynecology and Obstetrics (FIGO) stage, and risk status were the risk factors. The 12 immune-related lncRNAs correlated with patient age, pathological grade, and FIGO stage. Principal component analysis and functional annotation showed that the high-risk and low-risk groups separated better, and the immune status of the high-risk and low-risk groups differed. Nomogram and receiver operating characteristic (ROC) curves effectively predicted the prognosis of endometrial cancer. Additionally, age, pathological grade, FIGO stage, and risk status were all related to patient survival.

**Conclusion:**

We identified 12 immune-related lncRNAs affecting the prognosis of endometrial cancer, which may be useful as therapeutic targets and molecular biomarkers.

## 1. Introduction

Endometrial cancer is one of the most common malignant tumors in women. However, the mechanisms underlying endometrial cancer occurrence and development are unknown. Recent studies indicated that hypertension, diabetes, obesity, and estrogen replacement therapy are risk factors for endometrial cancer [1–4]. The main clinical manifestation of endometrial cancer is postmenopausal vaginal bleeding [5]. Currently, the main treatment methods are surgery, chemotherapy, radiotherapy, and neoadjuvant therapy [6–9]. In general, the prognosis of endometrial cancer is good; however, some patients with higher FIGO stages have a poor prognosis [10–12].

Long noncoding RNAs (lncRNAs) are more than 200 nucleotides in length and were recently shown to dynamically regulate gene expression at multiple levels [13–18]. LncRNAs play many roles such as in the growth and development of the body [19], as well as occurrence and development of many diseases, such as cardiovascular diseases [20, 21], neurodegenerative diseases [22], and malignant tumors [23].

Recently, tumor immunotherapy has gained attention. Immune cells and molecules are complex components in the tumor microenvironment and can promote or inhibit malignant tumor progression. The immune response in the tumor microenvironment has an important effect on tumor proliferation, invasion, and metastasis [24–29]. Many studies have focused on the immune response in the tumor microenvironment to regulate tumor progression. For example, in breast and colon cancer and melanoma models, by reprograming the tumor-associated macrophage population to a proinflammatory phenotype and increasing tumor immunogenicity, anti-MARCO monoclonal antibodies can inhibit tumor progression [30].

In this study, we explored the transcriptome data of endometrial cancer samples in the TCGA database. Immune-related lncRNAs coexpressed with immune-related genes were identified, and a prognostic nomogram was constructed to predict the overall survival rate. These lncRNAs show potential as therapeutic targets and molecular biomarkers of endometrial cancer.

## 2. Materials and Methods

### 2.1. Data Sources

Fragments per kilobase per million RNA sequencing data of 575 samples were downloaded from TCGA database (https://portal.gdc.cancer.gov/), with 23 normal samples and 552 endometrial cancer samples. Clinical information on endometrial cancer samples was downloaded from UCSC Xena (https://xenabrowser.net). We downloaded the gene sets IMMUNE_RESPONSE (systematic name: M13664), IMMUNE_SYSTEM_PROCESS (systematic name: M19817), GO_NEGATIVE_REGULATION_OF_ADAPTIVE_IMMUNE_RESPONSE (systematic name: M10422), GO_NEGATIVE_REGULATION_OF_IMMUNE_RESPONSE (systematic name: M15641), and GO_T_CELL_ACTIVATION_INVOLVED_IN_IMMUNE_RESPONSE (systematic name: M10714) from the gene set enrichment analysis (GSEA) database (https://software.broadinstitute.org/gsea/index.jsp). Immune-related genes were obtained from the IMMUNE_RESPONSE (systematic name: M13664) and IMMUNE_SYSTEM_PROCESS (systematic name: M19817) gene sets. Functional annotation was performed using the gene sets IMMUNE_RESPONSE (systematic name: M13664), GO_NEGATIVE_REGULATION_OF_ADAPTIVE_IMMUNE_RESPONSE (systematic name: M10422), GO_NEGATIVE_REGULATION_OF_IMMUNE_RESPONSE (systematic name: M15641), and GO_T_CELL_ACTIVATION_INVOLVED_IN_IMMUNE_RESPONSE (systematic name: M10714).

### 2.2. Acquisition of Immune-Related lncRNAs

By sorting and analyzing the transcriptome data of the endometrial cancer samples from TCGA database, the expression matrices of mRNA and lncRNA of endometrial cancer samples were obtained. Immune-related genes were obtained from the IMMUNE_RESPONSE (systematic name: M13664) and IMMUNE_SYSTEM_PROCESS (systematic name: M19817) gene sets. The immune-related gene expression matrix was obtained by collecting and analyzing the mRNA expression matrix and immune-related genes together using the limma package in *R* software. Finally, the limma package in *R* software was used to screen immune-related lncRNAs showing a coexpression relationship with immune-related genes, and an immune-related lncRNA expression matrix was obtained (correlation filter = 0.5; *p* value filter = 0.001).

### 2.3. Cox Regression, Survival, Independent Prognostic, and Clinical Correlation Analyses

By collecting and analyzing the clinical data, univariate (*p* value filter = 0.01) and multivariate Cox regression analyses were performed using the survival package in *R* software. The coexpression network was plotted using Cytoscape software (Cytoscape v. 3.7.2). The survival and survminer packages of *R* software were used to draw the survival curves of high- and low-risk groups. Risk curves were drawn using the pheatmap package in *R* software. Univariate- and multivariate-independent prognostic analyses were performed using the survival package in *R* software, and a multi-index receiver operating characteristic (ROC) curve was plotted using survivalROC package in *R* software to evaluate the accuracy of the constructed model. The ggpubr package in *R* software was used for clinical correlation analysis.

### 2.4. Immune and Stromal Scores

We used the limma and estimate packages in *R* software to calculate the immune scores and stromal scores of all samples. *R* software was then used to analyze the immune scores and stromal scores of different risk states.

### 2.5. Principal Component Analysis and GSEA

Principal component analysis was performed using the limma and scatterplot3d packages in *R* software. The 12 immune-related lncRNAs were functionally annotated using GSEA software (GSEA_4.0.2).

### 2.6. Prognostic Nomogram and Survival Analysis of Independent Prognostic Risk Factors

Using the foreign, survival, and caret packages in *R* software, 70% of the tumor samples were placed into the training group and 30% into the validation group. The rms, foreign, and survival packages in *R* software were used to perform multivariate Cox regression analysis of the training group and calculate the C-index of the training and validation groups. A nomogram was constructed using the rms, foreign, and survival packages in *R* software. The survival and timeROC packages in *R* software were used to construct multi-index ROC curves for the training and validation groups. The survival package in *R* software was used to perform Kaplan-Meier survival analysis for the training group.

### 2.7. Data Statistics

The statistical analysis was performed using *R* software (R-3.6.1), strawberry-Perl-5.30.0.1. *p* values <0.05 were considered to indicate statistically significant results.

## 3. Results

### 3.1. Immune-Related lncRNAs Associated with Prognosis

**Figure 1:**
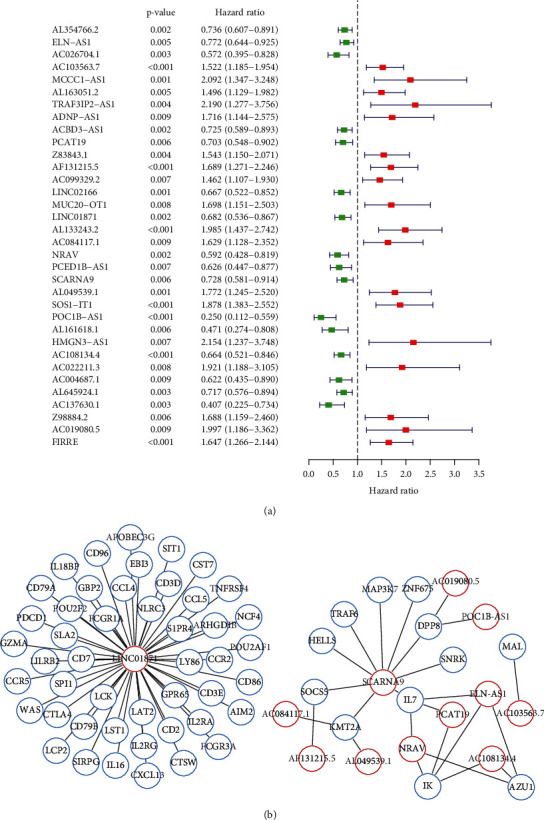
Immune-related lncRNAs associated with prognosis. (a) Forest plot of univariate Cox regression analysis. The *p* value, hazard ratio, and 95% confidence intervals of immune-related genes are shown. Red and green indicate risk-associated (HR > 1) and protective (HR < 1) lncRNAs, respectively. (b) Immune-related lncRNA-immune-related gene network. Green and red represent immune-related genes and 12 immune-related lncRNAs, respectively.

To identify immune-related lncRNAs associated with prognosis, 332 immune-related genes were selected from the GSEA data sets. Through data collection and analysis, we identified 137 immune-related genes and 363 immune-related lncRNAs showing a coexpression relationship with these immune-related genes. Next, univariate Cox regression analysis of all immune-related lncRNAs was performed, and a Forest plot was obtained (Figure 1(a)). As shown in Figure 1, green columns indicate protective lncRNAs [hazard ratio (HR) < 1], and red column indicates risk-associated lncRNAs (HR > 1). Next, 34 lncRNAs were subjected to multivariate Cox regression analysis, and 12 immune-related lncRNAs associated with prognosis were obtained (Table 1): ELN-AS1, AC103563.7, PCAT19, AF131215.5, LINC01871, AC084117.1, NRAV, SCARNA9, AL049539.1, POC1B-AS1, AC108134.4, and AC019080.5. Based on the median of risk scores, all endometrial cancer samples were divided into high- and low-risk groups: risk score = (expression level of ELN-AS1 × 0.229) + (expression level of AC103563.7 × 0.313) + (expression level of PCAT19 × −0.277) + (expression level of AF131215.5 × 0.252) + (expression level of LINC01871 × −0.357) + (expression level of AC084117.1 × 0.449) + (expression level of NRAV × −0.433) + (expression level of SCARNA9 × −0.339) + (expression level of AL049539.1 × 0.476) + (expression level of POC1B-AS1 × −0.758) + (expression level of AC108134.4 × −0.262) + (expression level of AC019080.5 × 0.899). Finally, an immune-related lncRNA-immune-related gene network was constructed using Cytoscape software (Figure 1(b)).

### 3.2. Survival Analysis and Risk Curves

**Figure 2:**
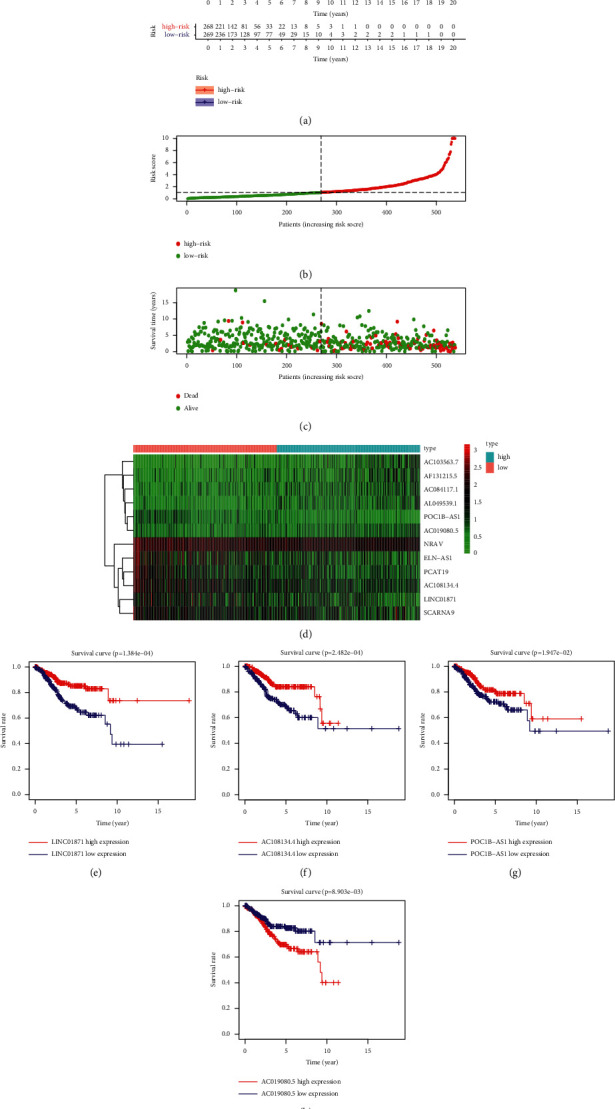
Survival analysis and risk curves. (a) Survival curve of endometrial cancer. Red and blue indicate high- and low-risk groups, respectively. (b) Risk curve of endometrial cancer. Red and green indicate high- and low-risk groups, respectively. (c) Scatter plot of different survival statuses of patients with endometrial cancer. Red and green dots denote patients that are dead or alive, respectively. (d) Hierarchical clustering of 12 immune-related lncRNAs. Differences in the expression levels of 12 immune-related lncRNAs in different risk statuses. ((e)–(h)) Survival curves of immune-related lncRNAs. Blue and red represent low- and high-expression groups, respectively.

To compare the survival rates of different risk statuses based on grouping results, survival analysis was performed, and survival curves were obtained (Figure 2(a)). As shown in Figure 2, the survival rate of the high-risk group was lower than that of the low-risk group. Risk curves for the high- and low-risk groups were obtained (Figures 2(b) and 2(c)). The results showed that the risk score of the high-risk group was higher than that of the low-risk group, and the survival time of the high-risk group was shorter than that of the low-risk group. Next, a heat map was drawn to compare the expression levels of the 12 immune-related lncRNAs with different risk statuses (Figure 2(d)). As shown in Figure 2, the expression levels of AC103563.7, AF131215.5, AC084117.1, and AL049539.1 in the high-risk group were higher than those in the low-risk group, and the expression levels of NRAV, ELN-AS1, PCAT19, AC108134.4, LINC01871, and SCARNA9 in the low-risk group were higher than those in the high-risk group. Survival curves were then plotted for the 12 immune-related lncRNAs to analyze the effects of these lncRNAs on survival (Figures 2(e)–2(h) and Supplementary Figure 1). As shown in Figure 2, the overall survival rate associated with LINC01871, AC108134.4, and POC1B-AS1 in the low-expression group was lower than that in the high-expression group. The overall survival rate associated with AC019080.5 in the low-expression group was higher than that in the high-expression group (*p* < 0.05).

### 3.3. Independent Prognostic and Clinical Correlation Analyses

**Figure 3:**
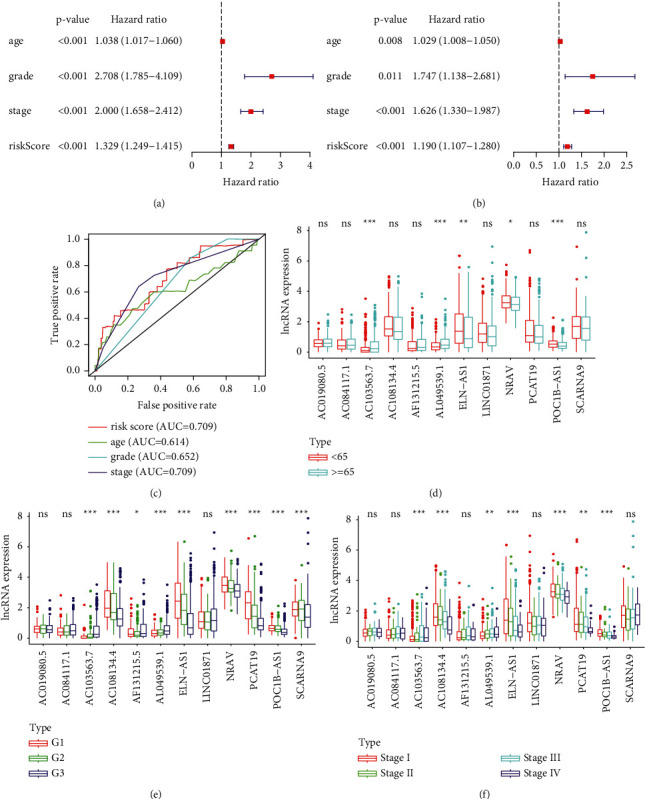
Independent prognostic analysis and clinical correlation analysis. (a) Forest plot of univariate independent prognostic analysis. The *p* value, hazard ratios, and 95% confidence intervals are shown. Red and green indicate risk-related (HR > 1) and protective (HR < 1) factors, respectively. (b) Forest plot of multivariate independent prognostic analysis. The *p* value, hazard ratios, and 95% confidence intervals are shown. Red and green indicate risk-related (HR > 1) and protective (HR < 1) factors, respectively. (c) Plot of multi-index ROC curve showing risk score (AUC = 0.709), age (AUC = 0.614), grade (AUC = 0.652), and stage (AUC = 0.709). (d) LncRNA expression levels in patients aged below (red) or above (blue) 65 years. (e) LncRNA expression levels in different pathological grades. (f) LncRNA expression levels in different FIGO stages. ^*∗∗*^*p* < 0.05, ^*∗∗*^*p* < 0.01, and ^*∗∗∗*^*p* < 0.001. ns, *p* > 0.05.

To analyze the impact of the patients' age, pathological grade, and FIGO stage on prognosis, we performed univariate and multivariate independent prognostic analyses of clinical data (Figures 3(a) and 3(b)). The results showed that patients' age, pathological grade, and FIGO stage were associated with prognosis and were risk factors for endometrial cancer. Older patients had a higher pathological grade and FIGO stage and poorer prognosis. We then constructed a multi-index ROC curve to evaluate the accuracy of all models (Figure 3(c)). As shown in Figure 3, risk score (area under the curve (AUC) = 0.709), age (AUC = 0.614), grade (AUC = 0.652), and stage (AUC = 0.709) indicated that the constructed model was accurate. Clinical correlation analysis was performed for the 12 immune-related lncRNAs and patients' age, pathological grade, and FIGO stage (Figures 3(d)–3(f)). As shown in Figure 3, AC103563.7, AL049539.1, ELN-AS1, NRAV, and POC1B-AS1 were associated with age, pathological grade, and FIGO stage; AC108134.4 and PCAT19 with pathological grade and FIGO stage; and AF131215.5 and SCARNA9 with pathological grade.

### 3.4. Immune Scores and Stromal Scores

**Figure 4:**
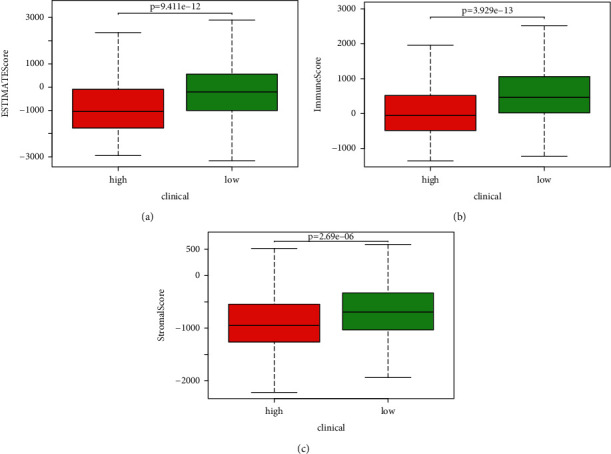
Immune scores and stromal scores. (a) Distribution of ESTIMATE scores of different risk statuses. Boxplot shows a significant difference between high- and low-risk groups (*p* < 0.05). (b) Distribution of immune scores of different risk statuses. Boxplot shows a significant difference between high- and low-risk groups (*p* < 0.05). (c) Distribution of stromal scores of different risk statuses. Boxplot shows a significant difference between high- and low-risk groups (*p* < 0.05).

To compare tumor microenvironment differences for different risk statuses using the ESTIMATE algorithm, the ESTIMATE, immune, and stromal scores of all samples were calculated. The results showed that the ESTIMATE scores ranged from −3166.978 to 3990.147, immune scores ranged from −1359.509 to 3614.677, and stromal scores ranged from −2224.623 to 860.431. Next, we drew box plots of these scores for different risk statuses (Figures 4(a)–4(c)). The average ESTIMATE scores, immune scores, and stromal scores of the high-risk group were lower than those of the low-risk group.

### 3.5. Principal Component and Gene Set Enrichment Analyses

**Figure 5:**
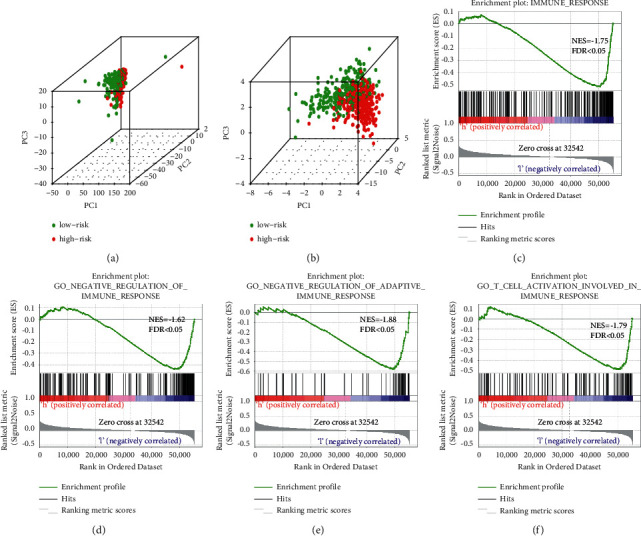
Principal component and gene set enrichment analyses. (a) Principal component analysis between low‐ and high‐risk groups based on all immune-related lncRNAs. Red and green indicate high- and low-risk groups, respectively. (b) Principal component analysis between low‐ and high‐risk groups based on 12 immune-related lncRNAs associated with prognosis. Red and green indicate high- and low-risk groups, respectively. ((c)–(f)) GSEA indicates significant enrichment in immune‐related phenotypes in low‐risk patients.

Principal component analyses of the expressions of all immune-related lncRNAs and 12 immune-related lncRNAs associated with prognosis were performed to determine whether there were differences in the distribution between the high- and low-risk groups (Figures 5(a) and 5(b)). As shown in Figure 5, the high- and low-risk groups showed better separations, and the immune statuses of these groups differed. Next, GSEA was performed on the 12 immune-related lncRNAs (Figures 5(c)–5(f)). As shown in Figure 5, compared with the high-risk group, the low-risk group was enriched in the gene sets IMMUNE_RESPONSE (systematic name: M13664), GO_NEGATIVE_REGULATION_OF_ADAPTIVE_IMMUNE_RESPONSE (systematic name: M10422), GO_NEGATIVE_REGULATION_OF_IMMUNE_RESPONSE (systematic name: M15641), and GO_T_CELL_ACTIVATION_INVOLVED_IN_IMMUNE_RESPONSE (systematic name: M10714). These immune-related lncRNAs may be associated with regulation of the immune response.

### 3.6. Prognostic Nomogram for Overall Survival Rate

**Figure 6:**
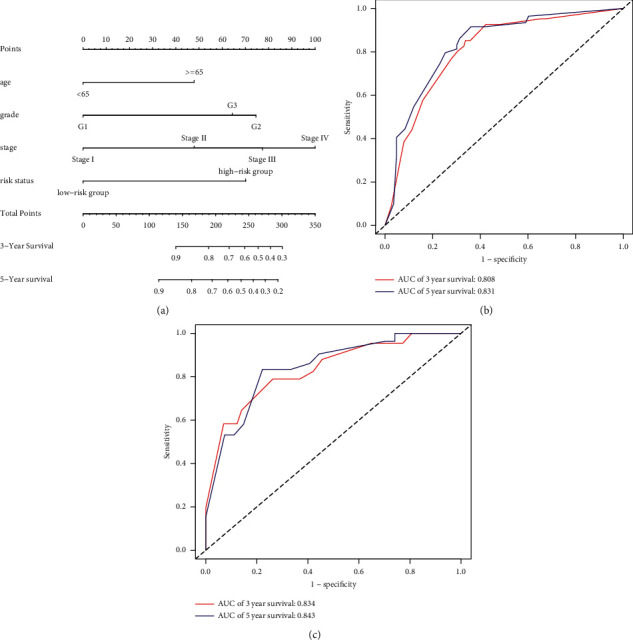
Prognostic nomogram for overall survival rate. (a) Survival nomogram (to use the nomogram, an individual patient's value is located on each variable axis, and a line is drawn upward to determine the number of points received for each variable value. The sum of these numbers is located on the Total Points axis, and a line is drawn downward to the survival axes to determine the likelihood of 3-year or 5-year survival). (b) Multi-index ROC curve of training samples. Red and blue indicate 3-year and 5-year survival, respectively. (c) Multi-index ROC curve of validation samples. Red and blue indicate 3-year and 5-year survival, respectively.

To further evaluate the effect of the model constructed based on the 12 immune-related lncRNAs on prognosis, we used the patient's risk status based on these lncRNAs as an independent risk factor affecting prognosis and divided all samples into training (70%) and validation (30%) groups. Multivariate Cox regression analysis using training samples was performed to analyze the effects of patient's age, pathological grade, FIGO stage, and risk status on prognosis (Table 2). The age, FIGO stage, and risk status were all associated with prognosis. A prognostic nomogram that integrated all significant independent factors affecting overall survival was constructed to predict the survival rate (Figure 6(a)). A multi-index ROC curve using the training samples was drawn to evaluate model accuracy (Figure 6(b)). The results showed the 3-year survival (AUC = 0.808) and 5-year survival (AUC = 0.831) rates. The C-index of the training group was 0.794 (standard error ± 0.029). Therefore, the prognostic nomogram showed good accuracy. Next, we used the verification samples to draw a multi-index ROC curve to evaluate the stability of the prognostic nomogram (Figure 6(c)). As shown in Figure 5, the 3-year survival (AUC = 0.834) and 5-year survival (AUC = 0.843) were larger than the AUC values of the training samples, and the C-index was 0.818 (standard error ± 0.036), which was larger than that of the training samples. Therefore, the prognostic nomogram showed good stability.

Age ≥65 years was compared to age <65 years; Grade 2 and Grade 3 were compared to Grade 1; Stage II, Stage III, and Stage IV were compared to Stage I; and high-risk group was compared to low-risk group. Regression coefficients, *p* values, hazard ratios, and 95% confidence intervals of the clinical characteristics are shown.

### 3.7. Survival Analysis for Independent Prognostic Risk Factors

**Figure 7:**
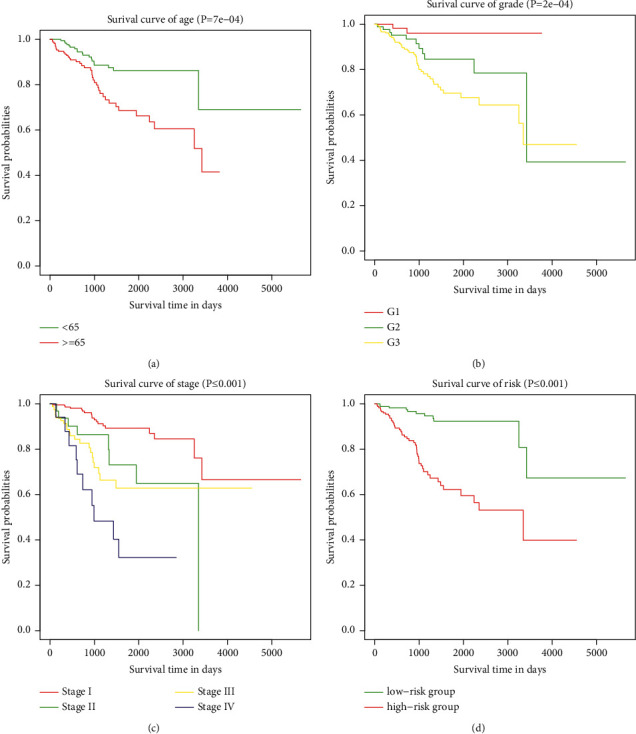
Survival analysis of independent prognostic risk factors. (a) Survival curves of patients below or above 65 years. (b) Survival curves of groups in Grade 1, Grade 2, and Grade 3. (c) Survival curves of groups in Stages I–IV. (d) Survival curves of patients in high- and low-risk groups divided based on 12 immune-related lncRNAs.

To analyze the effects of age, pathological grade, FIGO stage, and risk status on overall survival, each risk factor was subjected to Kaplan-Meier survival analysis, and survival curves were drawn (Figures 7(a)–7(d)). As shown in Figure 7, age, pathological grade, FIGO stage, and risk status based on the 12 immune-related lncRNAs affected patient prognosis (*p* < 0.05).

## 4. Discussion

Recent studies showed that the immune system has a dual effect on tumor progression. The immune components in the tumor microenvironment can both promote or inhibit the progression of malignant tumors [31]. Infiltrating immune cells, cytokines secreted by immune cells in the tumor microenvironment, and chemokines are involved in tumor progression [32–34]. In recent years, with widespread application of the immune checkpoint inhibitors PD-1/PD-L1 and CTLA-4, an increasing number of studies have been devoted to suppressing the progress of tumors by regulating the immune components in the tumor microenvironment [35–37]. For example, by regulating mitotic checkpoints and chromosome stability, TIF1*γ* inhibits tumor progression [38]. Recently, epigenetic regulation was shown to play a vital role in the occurrence and development of tumors, such as regulating tumor resistance, proliferation, metastasis, and epithelial-mesenchymal transition [39–44]. Interestingly, prognostic model based on epigenetic regulation was constructed to predict patient prognosis, such as a prognostic model based on nine ferroptosis-related genes (*ALOX15*, *CISD1*, *CS*, *GCLC*, *GPX4*, *SLC7A11*, *EMC2*, *G6PD*, and *ACSF2*) that could predict the prognosis of breast cancer [45]. LncRNAs also play an important role in epigenetic regulation; these molecules have complex functions and regulate the occurrence and development of malignant tumors through various mechanisms. For example, by inhibiting CUL4A-mediated LATS1 ubiquitination and increasing YAPS127 phosphorylation, lncRNA uc.134 can inhibit the progression of liver cancer [46]. In gastric cancer, low-expression lncRNA LINC00261 can inhibit tumor metastasis via regulating epithelial-mesenchymal transition [47]. In liver cancer, by degrading HNRNPA2B1 via ubiquitination, which reduces the stability of p52 and p65 mRNAs, and inhibiting the NF-*κ*B signaling pathway in hepatocellular carcinoma cells, lncRNA miR503HG inhibits tumor metastasis [48].

LncRNAs also affect tumor progression by regulating immune components in the tumor microenvironment. For example, in lung and breast cancer, lncRNA NKILA can upregulate the sensitivity of tumor-specific cytotoxic T lymphocytes and type 1 helper T cells to activation-induced cell death by inhibiting NF-*κ*B activity, thereby facilitating immune escape [49]. In colorectal cancer, by regulating SATB2, lncRNA SATB2-AS1 can regulate the transcription of type 1 helper T cell chemokines and immune cell density in the tumor microenvironment, thus suppressing tumor metastasis [50].

However, it is relatively unknown whether lncRNAs modulate endometrial cancer progression via the regulation of immune components in the tumor microenvironment. In the present work, the expression matrix of immune-related lncRNAs was analyzed by collecting immune gene sets from the GSEA database and information on endometrial cancer samples from TCGA database. Univariate and multifactorial Cox regression analyses were performed, and 12 immune-related lncRNAs were identified as having an important influence on endometrial cancer: ELN-AS1, AC103563.7, PCAT19, AF131215.5, LINC01871, AC084117.1, NRAV, SCARNA9, AL049539.1, POC1B-AS1, AC108134.4, and AC019080.5.

Among the 12 immune-related lncRNAs, PCAT19 level is downregulated in non-small cell lung carcinoma and regulates the development of prostate cancer [51–53]. By modulating the miR‐182/PDK4 axis, PCAT19 promotes the proliferation of laryngeal carcinoma cells [54]. SCARNA9 level is downregulated in cervical cancer [55]. Nevertheless, the specific functions of ELN-AS1, AC103563.7, AF131215.5, LINC01871, AC08417.1, NRAV, AL049539.1, POC1B-AS1, AC108134.4, and AC019080.5 in the tumor microenvironment remain unknown. These lncRNAs may affect the occurrence and development of endometrial cancer by regulating immune components in the tumor microenvironment. However, to understand the respective functions, further experimental validation is required. The 12 immune-related lncRNAs may represent new molecular biomarkers and therapeutic targets for endometrial cancer.

## 5. Conclusion

By sorting and analyzing the transcriptome information of endometrial cancer samples from TCGA database, we identified 12 immune-related lncRNAs. These molecules may play important regulatory roles in the occurrence and development of endometrial cancer and represent potential therapeutic targets. However, their specific roles and mechanisms require further experimental validation.

## Figures and Tables

**Table 1:** Multivariate Cox regression analysis of 12 immune-related lncRNAs.

LncRNA	Coefficient	HR	HR.95L	HR.95H	*p* value
ELN-AS1	0.229	1.257	0.963	1.640	0.092
AC103563.7	0.313	1.367	1.018	1.836	0.038
PCAT19	−0.277	0.758	0.564	1.018	0.065
AF131215.5	0.252	1.287	0.924	1.794	0.136
LINC01871	−0.357	0.700	0.560	0.873	0.002
AC084117.1	0.449	1.567	1.065	2.307	0.023
NRAV	−0.433	0.648	0.407	1.034	0.069
SCARNA9	−0.339	0.712	0.575	0.883	0.002
AL049539.1	0.476	1.610	1.041	2.488	0.032
POC1B-AS1	−0.758	0.469	0.203	1.083	0.076
AC108134.4	−0.262	0.770	0.552	1.073	0.122
AC019080.5	0.899	2.457	1.352	4.464	0.003

Regression coefficients, *p* value, hazard ratio, and 95% confidence interval of immune-related lncRNAs are shown.

**Table 2:** Multivariate Cox regression analysis of clinical characteristics.

Variable	Coefficient	HR	Lower.95	Upper.95	*p* value
Age ≥65 years	0.727	2.069	1.158	3.697	0.014
Grade 2	1.131	3.097	0.676	14.201	0.146
Grade 3	0.976	2.654	0.604	11.665	0.196
Stage II	0.726	2.067	0.881	4.847	0.095
Stage III	1.173	3.231	1.687	6.188	*p* ≤ 0.001
Stage IV	1.517	4.559	2.006	10.358	*p* ≤ 0.001
High-risk group	1.064	2.897	1.402	5.986	0.004

## Data Availability

The authors obtained RNA seq data from TCGA database (https://cancergenome.nih.gov/) and clinical information from the UCSC Xena (https://xenabrowser.net). All gene sets were downloaded from GSEA database (https://software.broadinstitute.org/gsea/index.jsp).

## Abbreviations

TCGA: The Cancer Genome Atlas
GSEA: Gene set enrichment analysis
HR: Hazard ratio
NES: Normalized enrichment score
FIGO: The International Federation of Gynecology and Obstetrics
ROC: Receiver operating characteristic.

## Supplementary Materials

Supplementary Materials Supplementary Figure 1: survival curves of immune-related lncRNAs. As shown in Supplementary Figure 1, the overall survival rate associated with ELN-AS1, PCAT19, NRAV, and SCARNA9 in the low-expression group was lower than that in the high-expression group. The overall survival rate associated with AC084117.1, AC103563.7, AF131215.5, and AL049539.1 in the low-expression group was higher than that in the high-expression group (*p* < 0.05). Supplementary Figure 1 ((A)–(H)): survival curves of immune-related lncRNAs. Blue and red represent low- and high-expression groups, respectively.
