# Supplementary material for: An Immune-Related Long Noncoding RNA Signature as a Prognostic Biomarker for Human Endometrial Cancer
Source: J Oncol. 2021 Dec 10;2021:9972454. doi: 10.1155/2021/9972454 (PMC8683168; doi:10.1155/2021/9972454)
Supplement: Supplementary Materials — Supplementary Figure 1: survival curves of immune-related lncRNAs. As shown in Supplementary Figure 1, the overall survival rate associated with ELN-AS1, PCAT19, NRAV, and SCARNA9 in the low-expression group was lower than that in the high-expression group. The overall survival rate associated with AC084117.1, AC103563.7, AF131215.5, and AL049539.1 in the low-expression group was higher than that in the high-expression group (p < 0.05). Supplementary Figure 1 ((A)–(H)): survival curves of immune-related lncRNAs. Blue and red represent low- and high-expression groups, respectively. [file 9972454.f1.docx]

**Supplementary Data**

Supplementary Figure 1. Survival curves of immune-related LncRNAs


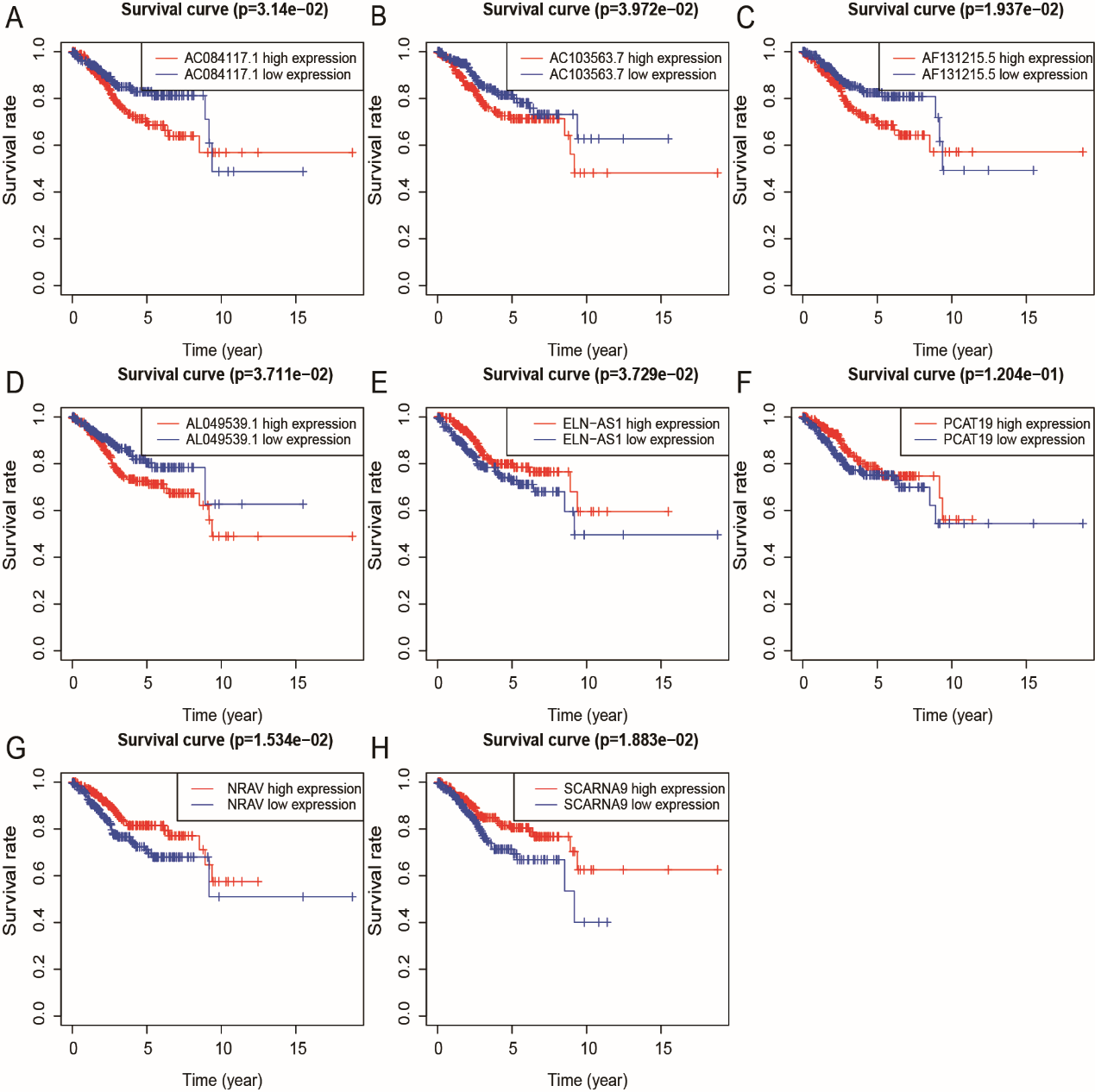


Supplementary Figure 1. (A-H) Survival curves of immune-related lncRNAs. Blue and red represent low and high expression groups, respectively
